# Validating self-administration as an agile modality for high-frequency diet quality data collection

**DOI:** 10.1371/journal.pone.0317611

**Published:** 2025-06-25

**Authors:** Rhys Manners, Anna W. Herforth, Maria Delfine, Rosil Hesen, Didier Nkubito, Karin Borgonjen-van den Berg, Eric Matsiko, Marguerite Niyibituronsa, Betül T. M. Uyar, Elise F. Talsma

**Affiliations:** 1 International Institute of Tropical Agriculture, Kigali, Rwanda; 2 Division of Human Nutrition and Health, Wageningen University and Research (WUR), the Netherlands; 3 Strategic Development and Research Group, Kigali, Rwanda; 4 University of Rwanda, Kigali, Rwanda; 5 Rwanda Agriculture and Animal Resources Development Board (RAB); Bangladesh Institute of Social Research (BISR) Trust, BANGLADESH

## Abstract

Data collection of diet quality is important to estimate global dietary transitions affecting public health. Mobile-phone based tools can provide a low-cost and rapidly deployable modality to complement enumerator-collected data. This study validated self-administration of the Diet Quality Questionnaire using mobile phones, comparing accuracy against enumerator administration, measuring both against an observed benchmark. Quantitative dietary intake data were gathered from 308 participants in northwest Rwanda, using a weighed food record. Intake data were used to calculated ‘observed’ responses to the questionnaire, half the participants responding to enumerators, and the other half using a mobile-administered version of the questionnaire. After filtering for low quality data, agreement in observed and reported responses, for all questionnaire questions, were statistically compared. Agreement rates (observed-reported) of self-administered and enumerated responses were high (91% vs 95%, p = 0.05), respectively. Agreement was significantly lower for the mobile-administered modality among older and lower income respondents (by about 5 percentage points), with no significant differences by gender or time of response. Mobile-administration cost approximately USD 0.70 per response, versus the marginal cost of USD 0.79 for the enumerator-administered. Our results confirm self-administered reporting by mobile-phone as a valid, low-cost method for collecting dietary data, with only marginal (yet significant for some subgroups) differences in agreement rates, compared to enumerated data. Data collection by mobile phone represents an agile complement to enumerated collection, administrable in the absence of existing survey platforms; and provides a useful option for high-frequency data collection to monitor dietary dynamics in target sub-populations.

## Introduction

As of 2023, hunger affected 713–757 million people, with 2.3 billion experiencing moderate to severe food insecurity, and 2.8 billion unable to afford a healthy diet [1]. Sub-Saharan Africa accounts for a large fraction of these global statistics. Factors contributing to this situation include poverty, climate change impacts on agriculture, compounded by war, trade restrictions, and the COVID-19 pandemic [2–4]. The worsening situation emphasises the need for food systems transformation to improve food security and nutrition [5–7].

Innovative shifts in nutrition programmes to support these transformations require simultaneous improvements in data collection pipelines, generating data in relevant and actionable timescales. Data and data collection systems in low and lower-middle income countries (LMICs), although rapidly improving, may not be able to support required dataflows [8]. The mobile-phone revolution taking place across most LMICs presents new possibilities for novel, economic, and scalable data collection mechanisms to encourage improved data flows and [near] real-time monitoring of systems and support dynamic decision-making [9]. Mobile-phone based collection systems are already deployed and assessing the status and changes in food systems [10–12].

Manners et al. [13] used a mobile-phone based tool to pilot a lean and low-cost data collection system for monitoring diet quality in Rwanda. This work consisted of deployment of the Diet Quality Questionnaire (DQQ) [14], a low-burden 5-minute tool for dietary assessment. The DQQ allows for calculation of more than 50 indicators of diet quality, based upon responses to the questionnaire’s 29 yes/ no questions [15]. Using a well-established and ubiquitous mobile-phone platform (unstructured supplementary service data: USSD), Manners et al. [13] sent the DQQ directly to respondents. In 52 weeks of data collection (August 2021-August 2022), the system collected self-administered responses from more than 80,000 unique respondents, collecting around ~1,800 responses per week. The success of the pilot suggested a viable alternative modality for collecting the DQQ, complementing enumerated data collection carried out within the Gallup World Poll and DHS.

However, questions regarding the accuracy and reliability of self-administrated data are prevalent [e.g., 16] and are relevant to this novel, yet unvalidated data collection system in Rwanda. Inaccuracies of self-administered data may be intentional (for malicious purposes or gaming of the system), or unintentional (lack of understanding of questions) [9,17–19]. Enumerated data collection also may be subject to enumerator and social desirability biases [20–23]. While both self-administered and enumerated data collections may have errors, the latter are often costly and time consuming [13]. Understanding how, if at all, a method of data collection affects responses can help to create validated and reliable multi-modality agile data collection systems.

The aim of this study was to investigate how the modality of data collection affects responses to the DQQ and diet quality indicators. We compared the accuracy of responses to the DQQ and diet quality indicators across two modalities: self-administered and enumerator administered, against an observed benchmark generated from a weighed food record. We also sought to develop a mechanism for identifying and filtering out low quality responses using a minimum quality threshold [18].

## Materials and methods

### Study area

**Fig 1:**
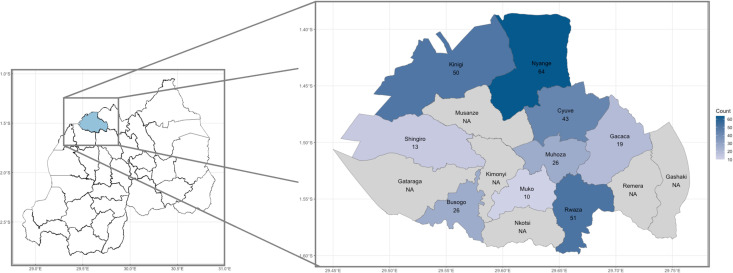
The study site in the District of Musanze and its constituent sectors in northwest Rwanda. Initial participant numbers per sector are highlighted with both colour palette and overlayed numbers.

The study was performed from July-August 2023 in the Musanze district of northwest Rwanda (Fig 1). Musanze is a largely rural and peri-urban highland region, characterised by high rainfall and volcanic rich soils, with agriculture dominating economic activities.

Musanze was selected due to the relatively high participation rate in the previous conducted pilot study [13], the ease with which it can be traversed, and concentrated population clusters to reduce implementation costs. Data were collected across 9 of Musanze’s 15 constituent sectors (sub-district administrative boundaries) (Fig 1).

### Study structure

The study was structured to generate comparable responses to the DQQ across two reported collection modalities: i) enumerator administered (from here on “enumerator”) and ii) self-administered using mobile phones (from here on “mobile-phone”). Reported responses (enumerator or mobile-phone) were compared to observed consumption from a weighed food record (WFR) benchmark.

Data were collected across two days. On day one, respondents from both groups were visited by an enumerator, where consumption was recorded via a weighed food record. On the second day, the ‘enumerator’ group was visited by a different enumerator, who administered the DQQ and a 24-hr recall. The ‘mobile-phone’ group administered the DQQ using their mobile-phones through an unstructured supplementary service data (USSD) system. USSD is a text-based system that functions on both smart and basic mobile-phones. Following submission, the mobile-phone group was visited by an enumerator, who administered a 24-hr recall. More details are provided in section 2.5 (Data Collection).

### Sample

To determine a sample size, we conservatively assumed a medium effect size (0.35) in the differences between the observed-DQQ benchmark and the two groups (enumerator and mobile-phone), setting the power at 80% and significance level at 5%, providing a sample size of 130 (per group). We increased this to 150 (per group), assuming a 15% non-response rate from the mobile-phone group and to account for participant dropouts [13]. This sample size is consistent with similar studies [24,25].

We endeavoured to perform the WFR with both male and female respondents, aiming for a 50−50 ratio in responses. Although the two groups could not be stratified representatively across gender, age, or economic groups, we aimed to derive inferences on any differences in responses across such groups.

### Participant recruitment

An interactive voice response (IVR) messaging system was deployed to contact potential participants. The IVR system communicated with individuals who had previously participated in the piloting of the self-administered deployment of the DQQ (see [13]. We do not believe that prior use of the mobile-phone system would have any biassing effect upon respondents, due to the ubiquity of USSD- based services in Rwanda (e.g., for financial and agricultural services). The IVR message provided information (via a pre-recorded audio message in Kinyarwanda) on the study. Consenting respondents were invited to provide information on their gender, age, location, economic group (*ubudehe,* all citizens of Rwanda were grouped into an economic category based upon their assets and income – categories are graded from 1 (the poorest) to 4) [26], and whether they were interested in participating in the study.

From June 18-July 9, 2023, 632 consenting participants were onboarded. From this cohort, 21 individuals participated in a pilot data collection; these individuals were removed from the group for subsequent sampling. The remaining 611 participants were split into the two study groups (enumerator and mobile phone) using a random number generator. To ensure that our two groups were statistically homogeneous, Chi^2^ tests were used to compare the respondents within both groups. Three days before visiting the households, we randomly communicated with potential respondents to confirm their interest in participating; participation was further confirmed 24 hours before the data collection with each respondent. This process was repeated until the required number of respondents for each group was achieved.

Recognising that participation in the study was invasive and time intensive, in particular the WFR, we compensated participants with a payment of $5 in the form of mobile money, a USSD based digital payment system.

### Data collection

#### Enumerator training.

Enumerators were trained for 5 days on the use and recording of data in KoboToolbox (a digital data collection app), implementing a WFR data collection, DQQ data collection, and 24-hr recall data collection. Data collection piloting was performed for 1.5 days, to monitor enumerators and address any questions and resolve data collection problems.

#### Weighed food record.

To perform the WFR, enumerators arrived at the respondents’ household by 6am and stayed until 8 pm, recording the preparation and consumption of all meals and individual foods consumed throughout the day. Enumerators were instructed to follow respondents throughout the 14-hour window, shadowing, but not interfering, with the respondent’s daily routine. To ensure recording of meals and foods consumed before 6am and after 8 pm, a 24-hr recall was implemented by a different enumerator on the second day of data collection. Foods and meals consumed outside of the WFR data collection window, but declared in the 24-hr recall were included in the observed DQQ (See Observed DQQ Benchmark).

On arrival at the household, enumerators recorded the respondent’s gender, age (18–24, 25–34, 35–44, > 44) economic group (ubudehe category 1:4), education level (no school, primary school, secondary school, post-secondary school, adult education), and telephone numbers. This information was used as a cross reference to ensure respondents on Day 1 were the same as those in Day 2. Enumerators asked respondents whether they would be available all of Day 1 and briefly available on Day 2. Enumerators were also instructed to ask if the respondents would cook their own food. In cases where cooking would be supported by other family members, enumerators asked if the cook could participate in the study.

Enumerators recorded all foods and drinks consumed during the 14-hour data collection window. They recorded the ingredients as they were prepared, the weight of the empty cooking utensil (e.g., pot, bowl), the weight of each ingredient before being cooked, the weight of the amount served, the weight of the plate, and the weight of any leftovers (i.e., food not consumed by the respondent during the meal). All weights were tared and recorded using a digital kitchen scale (SF-400) with a precision of 1g and a maximum recording weight of 10 kg. Data were collected for all ingredients within dishes and single food items (e.g., a fruit consumed alone, a meat-brochette, or a soda). Enumerators recorded whether the item was an ingredient in a dish (recording the name of the dish) or a single food item.

### Diet Quality Questionnaire

#### Enumerator administered.

On Day 2, the DQQ was administered by a different enumerator from the WFR, to avoid potential enumerator biassing effects. Enumerators visited the respondent and collected the DQQ by reading the questionnaire exactly as written, without further probing [27]. Collected DQQ data were complemented by gender, age, economic group, education level, and telephone number data to cross reference against the data collected in Day 1. This cross referencing was performed during data cleaning, with discrepancies (e.g., non-consistent respondents: male, aged 25–34 on Day 1 and female aged above 44 on Day 2) dropped.

#### Mobile-phone administered.

On Day 2, respondents in the mobile-phone group received the DQQ via USSD by 6am. In this group, their telephone number was shared with the project partner, VIAMO, who sent an introductory SMS message to the respondent at 6am. This message instructed the respondent on how to initiate the DQQ. Following these instructions, the DQQ was displayed on their phones. Like the enumerator-administered group, the mobile-phone DQQ was complemented by collection of the same socio-economic information for later cross-referencing. The completed DQQ was automatically sent to VIAMO’s server. Following submission, VIAMO instructed enumerators they could visit the household. Enumerators were instructed not to visit before, or during, the answering of the DQQ to limit any enumerator influenced biases. Enumerators visited the household within 2 hours of the DQQ submission, almost exclusively before lunchtime.

Following completion of the DQQ, the methods for data collection across both groups were identical.

#### 24-hr recall.

Following the collection of the DQQ, respondents from both groups answered a multiple-pass 24-hr recall questionnaire [27]. As part of this, respondents were invited to recall all food and drinks consumed between waking and sleeping the previous day (Day 1). Enumerators also asked about preparation and consumption methods. Respondents were also asked to estimate the weight of the items consumed. When food items were available at home, enumerators used a digital kitchen scale to measure their weight. In cases where items were not available, alternative food items (such as maize flour or water) were used and conversion factors generated (weight-to-weight, volume-to-weight, standard serving sizes, and waste factors). For waste factors and composite dishes consumed out of home, recipes and waste factors per serving size were collected from three different markets and local restaurants in the Musanze area.

The 24-hr recall data were collected using Wageningen University and Research’s proprietary data collection app- ‘Catch-24’. This application allows enumerators to easily record all items, time of consumption, and add additional information.

### Consent and ethical approval information

Research approval was provided by the National Institute of Statistics Rwanda (No: 0127/2023/10/NISR) and the Ministry of Local Government (No: 0431/07.03), with ethical approval provided by the Rwanda National Research Ethics Committee (No: 150/RNEC/2023). All respondents provided written consent for their participation in the onboarding stage and again prior to the weighed food record collection.

### Data processing and preparation

All data were collected using KoboToolbox and ODK-based forms on enumerators’ tablets. As all data were collected digitally, submissions (WFR, DQQ, and 24-hr recall) were reviewed on a nightly basis. This afforded the opportunity to discuss problems immediately with enumerators during daily debriefs. For the WFR, we checked if the data collection procedure was followed (e.g., recording ingredients correctly for dishes and individual food items) and identifying erroneously recorded amounts (e.g., large amounts of a given ingredient/food item). For the 24-hr recall we performed a similar check, reviewing recorded items and amounts and flagging potential errors with enumerators. Enumerators received follow-up training and were monitored by a member of the research team on the following. All data from respondents affected by these errors were dropped from the study.

### Observed DQQ benchmark

We coded the WFR observations for all respondents, supplemented by the quantitative 24-hour recall, into an ‘observed DQQ’, taken as the comparative benchmark of the study. Using data collected from the WFR we coded all individual food items or ingredients consumed (in amounts above 15g) into the DQQ 29 questions. The DQQ comprises 29 questions, each representing a food group, with a number of sentinel foods per food group. Based upon observed consumed foods we assigned them to one of the 29 DQQ food groups (e.g., if enumerators observed respondents consuming beans, we converted this to a ‘yes’ for question 4 (legumes) of the DQQ) [28,29]. Recognising that respondents may consume before or after the enumerator visit, we supplemented the WFR data with that from the 24-hr recall. To do this, we filtered all data reported in the 24-hr recall consumed after 19:30, or before 06:00, and performed a similar coding procedure. We selected 19:30 as more than 90% of WFR were submitted by 20:00. We selected this buffer, recognising that enumerators may be tired and not be so observant before leaving and to take account of consumption after the enumerator had left the household.

### Analysis

#### DQQ question responses.

**Fig 2:**
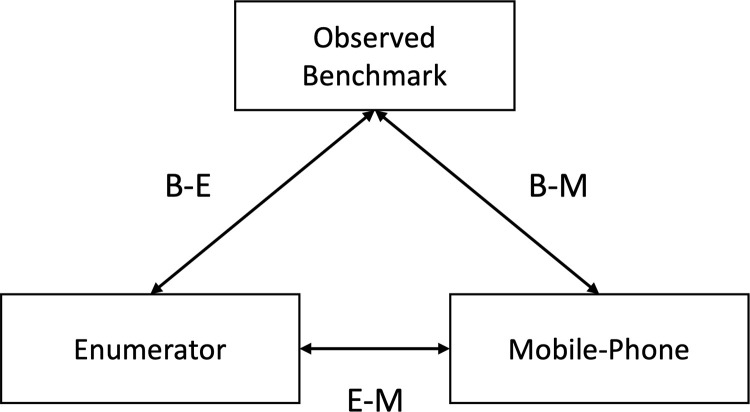
Triangulated approach for comparing success reported modalities of DQQ data collection, compared to an observed benchmark.

We compared the reported DQQ responses from both modalities (enumerator; mobile phone), against the observed benchmark (WFR) and calculated their relative accuracy in a triangulated approach (Fig 2). For B-E (benchmark-enumerator) and B-M (benchmark-mobile phone), we estimated the levels of agreement in percentage terms (accuracy) between observed and reported responses for each DQQ question, generating three metrics per question: i) percentage agreement – where both observed and reported have the same response; ii) false negative rate- where the observed DQQ recorded consumption, but the reported did not; iii) false positive rate- where the observed DQQ recorded no consumption, but the reported did. To test the modality relationship (E-M; Fig 2) we used a Mann-Whitney U test to compare the percentage agreement, across the 29 question. We considered that a p-value <0.05 for the E-M relationship would indicate the superiority of one method over the other for collection of the DQQ. We also calculated a delta value, in percentage agreement, across both modalities per DQQ question. Following Uyar et al. [30], we considered that if one modality had a delta greater than 10, it would suggest a practically important improvement of one modality for a given question.

#### Socio-economic characteristics.

To complement the analysis at the DQQ question level, we evaluated potential effects of socio-economic variables (gender, age, education level, and economic group) on agreement levels. Although the respondents in each modality group could not be stratified representatively, we aimed to derive inferences on how socio-economic factors affect responses between modalities. For each respondent, we calculated the percentage agreement between their reported and observed responses to the 29 questions of the DQQ. We used non-parametric statistics due to the non-normal distribution of the percentage agreement values, confirmed with a Shapiro-Wilk test, and the ordinal nature of the socio-economic variables. Mann-Whitney U test were applied for binary comparisons (gender) and the Kruskal-Wallis test for multiple group variables (age, education level, and economic group). Post-hoc analysis was conducted using Dunn’s test with Bonferroni correction for multiple comparisons. These tests were applied to test if significant differences could be observed within modalities (e.g., female and male respondents in the mobile-phone administered group) or between modalities (e.g., respondents aged 35–44 in both groups). We set the significance level at p < 0.05 for all tests.

#### Time of day.

We hypothesised that responses may be more accurate if data were collected in the morning, so that participants could recall consumption from the previous day without having consumed much food in the day of the interview. We analysed agreement between DQQ (both enumerator administered, and mobile phone administered) by hour of completion and period of day (morning, afternoon, or evening). This temporal information was selected due to their collection in Manners et al [13] and their reported impacts on response quality [31].

#### Diet quality indicators.

The DQQ supports the calculation of more than 50 indicators of diet quality [32]. We focussed this analysis on seven indicators, which are core summary measures of diet quality and used by the Food Systems Countdown Initiative [33]. Indicators were calculated for all respondents using R codes available on the Global Diet Quality Project website (https://www.dietquality.org/calculator). These indicators come in two types: prevalence indicators which include Minimum Dietary Diversity for Women (MDD-W), a proxy indicator of micronutrient intake among women aged 15–49 years [29,34]; All-5, an indicator reflecting proportion of the population consuming all five food groups typically recommended for daily consumption in food-based dietary guidelines; Zero vegetable or fruit consumption, and Soft drink consumption [35,36] is the proportion of respondents who consumed the relevant foods for a binary indicator, and score (Dietary Diversity Score (DDS), Non-Communicable Disease (NCD)-Protect, NCD-Limit) is the mean from all respondents where indicators have a range based upon consumption on a number of different good groups [35,37].

We tested the individual prevalence values using a McNemar test, and all individual scores using a paired Mann-Whitney test. We also explored whether the modality of DQQ collection had a statistically relevant impact, comparing observed and reported responses (B-E and B-M). We did not compare if diet quality indicators were statistically different between modalities (E-M) as this may not suggest the impacts of the modality, just variance in the respondents within the groups.

#### Data quality protocol.

Measuring and improving the quality of data is a minimum requirement to ensure trust in data generation and facilitate data-driven decision-making [38]. We applied a simple data quality threshold [18] to the responses, we tested metrics of data quality to identify ‘low quality’ responses that could be flagged for removal to improve data robustness and reliability. Manners et al. [13] identified a number of respondents who may have provided false responses to ‘game’ the system for the incentive (e.g., answering all questions ‘yes’ or answering randomly yes/no/yes/no).

We calculated the mean number of ‘yes’ responses to the DQQ for each modality, independently. We assumed that most responses should fall within a 95% range (or two standard deviations) of this mean value (number of yes responses), using a rounded upper bound of the 95% range as the threshold for data to be flagged as ‘low quality’. Author knowledge of the context suggests that individuals may be observed to consume minimal food groups (e.g., < 2) on a given day; therefore the threshold was only implemented on the upper end. For those individuals who fell above the threshold, their responses were flagged for removal.

#### Cost.

Manners et al. [15] reported that the financial benefits of mobile phone administration of the DQQ were considerable. We report on the costs, per DQQ response, for both modalities. We collected information on the cost of deploying enumerators to the field for collection of the DQQ; for the mobile-phone approach, we recorded the system maintenance and administration costs. We excluded incentives from this cost calculation as they were unrealistically elevated ($5) for both approaches.

All data were processed and analysed using R [39]

## Results

### Respondents

**Fig 3:**
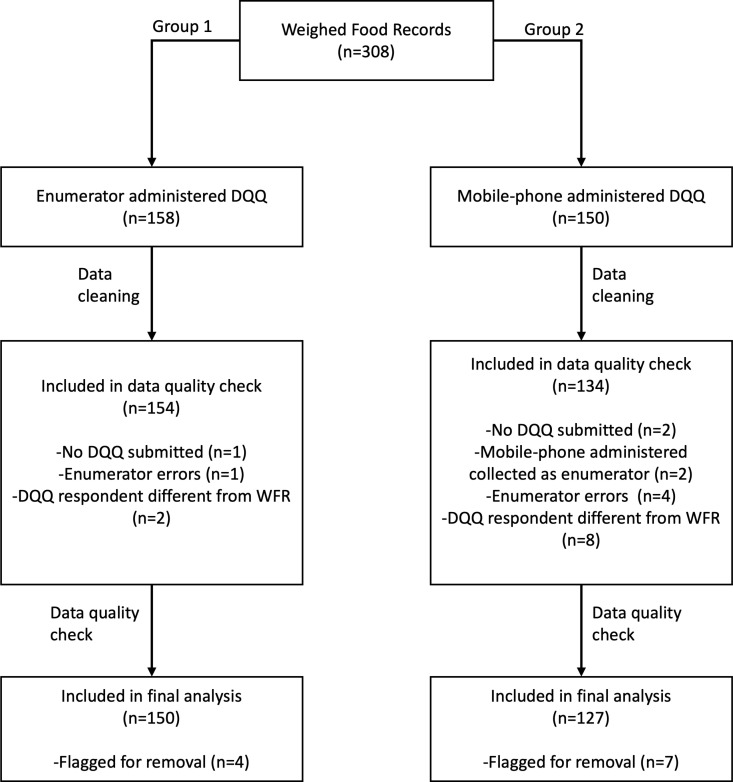
Distribution and removal of respondents from study and across study groups.

We visited 308 respondents (Fig 3), split across the enumerator administered group (n = 158) and mobile-phone administered (n = 150). Data were collected from 158, rather than 150, respondents in the enumerator administered group due to how enumerators were sent to the field in blocks and the enumerator administered group required an extra day of data collection, resulting in the sample size exceeding requirements.

Data from 20 respondents were removed from the study (Fig 3): 3 respondents as no DQQ data were collected; 2 mobile phone respondents were erroneously contacted as enumerator respondents; 5 were dropped due to enumerator errors, with enumerators failing to fully understand the methodology or who visited the respondent before completing the mobile-phone based DQQ, voiding the responses; 10 respondents were dropped upon discovery that the WFR and DQQ were not done by the same person (using cross-referenced data on age and gender). We retained responses from 288 respondents, distributed across the two groups (enumerator: 154 and mobile-phone: 134). These responses were then analysed for data quality.

### Data quality

We identified 4 respondents from the enumerator group and 7 from the mobile-phone group who exceeded the data quality threshold. Table 1 presents the levels of agreement, false negative, and false positive responses before and after excluding respondents. Before removal of these respondents, agreement levels were 95.1 (±5.78) for the enumerator groups and 89.4 (±8.64) for the mobile phone group, with agreement being significantly higher for the enumerator group (*p = 0.004*) and marginally significantly higher false positive (*p = 0.03*).

**Table 1:** Agreement between observed and reported DQQ responses. Pre-data quality check presents the agreement of DQQ responses for enumerator (n = 154) and mobile-phone (n = 134) respondents compared to observed responses. Post-data quality check presents the agreement of DQQ responses for enumerator (n = 150) and mobile phone (n = 127) respondents following removal of respondents who exceeded the data quality threshold. Agreement rates (reported versus observed) are average rates for all respondents, across the 29 DQQ questions.

	Pre-Data Quality Check			Post Data Quality Check		
	Enumerator Administered Mean (sd)	Mobile-Phone Administered Mean (sd)	*Statistical difference in agreement across DQQ questions (P-*Value)	Enumerator Administered Mean (sd)	Mobile-Phone Administered Mean (sd)	*Statistical difference in agreement across DQQ questions (P-*Value)
Mean agreement (%)	95.1 (5.78)	89.4 (8.64)	0.01^**^	95.4 (5.78)	91.1 (9.03)	0.05
False Negative (%)	2.68 (4.35)	5.12 (6.66)	0.05	2.71 (4.35)	4.94 (7.04)	0.18
False Positive (%)	2.23 (2.35)	5.53 (5.13)	0.03^*^	1.89 (2.38)	3.94 (4.39)	0.27

* *p* < 0.05,

** *p* < 0.01,

*** *p* < 0.001

Removal of flagged respondents improved agreement rates between observed and reported responses to the questions for both groups (B-E and B-M), but to a greater extent in the mobile-phone group. Following removal, mean response agreement in the enumerator administered group was 95.4% (±5.78), whereas mobile-phone administered respondents’ agreement was 91.1% (±9.03) (Table 1). Application of Mann-Whitney U test showed the between modality differences (E-M) to be marginally insignificant for mean agreement (*p* = 0.05).

### Respondent characteristics

We retained responses from 160 women (57.8%) and 117 men (42.2%). 230 (83.0%) respondents were under the age of 44, with the largest cohort (39.7%) aged 25–34. Most respondents were in the middle economic groups (2 and 3), with no respondents from the wealthiest group (4) and only 9.0% of respondents coming from the poorest group (1). The majority (54.5%) of respondents received primary school level education, 30.0% completed secondary school, and 11.6% of respondents received no formal education. We observed no noteworthy differences in the demographic composition of the two groups (Table 2).

**Table 2:** Demographic summary of retained respondents in both groups. Information generated following data cleaning removal of low-quality respondents.

	Enumerator Administered DQQ (n = 150)		Mobile-phone Administered (n = 127)	
	n	%	n	%
**Gender**				
Female	82	54.7	78	61.4
Male	68	45.3	49	38.6
**Age**				
18-24	23	15.3	21	16.5
25-34	63	42.0	47	37.0
35-44	38	25.3	38	29.9
Above 44	26	17.3	21	16.5
				
**Ubudehe** **(Economic Group)** ^*^				
1	10	6.7	17	13.4
2	87	58.0	57	44.9
3	51	34.0	51	40.2
4	–	–	–	–
Don’t know	2	1.3	2	1.6
**Highest Completed Education Level**				
No answer	3	2.0	1	0.8
No school	17	11.3	15	11.8
Primary	83	55.3	68	53.5
Secondary	43	28.7	40	31.5
Post Secondary	3	2.0	1	0.8
Adult Education	1	0.7	2	1.6

* Group 1: Very poor and vulnerable individuals who are unable to support themselves without assistance; Group 2: Individuals without fixed employment, living in rented accommodation and able to eat once or twice a day; Group 3: Employed individuals with their own small businesses. Group 4: Employed individuals who are government employees or owners of their own business. Adapted from Alexis (2023).

### Observed-reported agreement on DQQ questions

**Fig 4:**
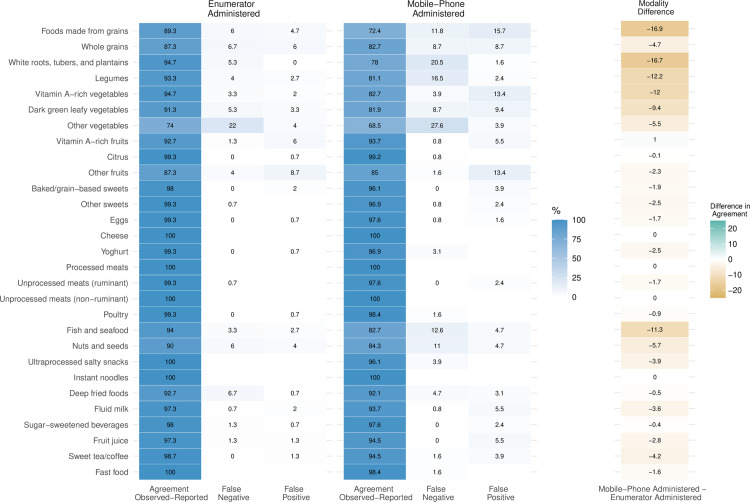
Agreement of reported and observed responses to the 29 questions of the Diet Quality Questionnaire from enumerator (n = 150) and mobile-phone (n = 127). Errors (false negative and false positive) and difference in agreement across reporting modalities are presented as a percentage points difference in agreement for each question.

We disaggregated the summarised results of Table 1 to explore agreement, false negative, and false positives rate for each food group (DQQ question) (Fig 4). For both modalities, ‘Other vegetables’ had the lowest agreement levels (enumerator: 74%; mobile-phone: 69%), tending towards false negative responses, while ‘other fruits’ (enumerator: 87%; mobile-phone: 85%) tended toward false positives, and ‘Foods made from grains’ (enumerator: 89%; mobile-phone: 72%) and ‘Whole grains’ (enumerator: 87%; mobile-phone: 83%) had similar false negatives and false positives. Modality differences (E-M) above our predetermined threshold (10 percentage points) were observed for ‘Foods made from grains’ (16.9 percentage points), ‘White roots, tubers, and plantains’ (16.7), ‘Legumes’ (12.2), ‘Vitamin A-rich vegetables’ (12), and ‘Fish and seafood’ (11.3 percentage points).

In Table 3, we present the nuanced and, in some cases, significant differences in percentage agreement when considering gender, age, education, and ubudehe (economic group) across modalities. We observed no significant differences in socio-economic variables within a modality group, but significant differences in socio-economic variables between modalities. Significantly higher agreement levels in both female (p=<0.000) and male (p = 0.01) respondents were observed from enumerator administered respondents. Respondents educated to primary school level in the mobile phone group had significantly lower levels of agreement compared with those in the enumerator administered group (p=<0.000). We also observed that older respondents (>35 years old), in the mobile phone administered group, showed significantly lower percentage agreement compared to their counterparts in the enumerator administered group. Middle income respondents (category 2) in the mobile phone group also provided significantly lower percentage agreement levels (p=<0.00), with the poorest category (1) providing only marginally insignificant responses (p = 0.07).

**Table 3:** Comparison of agreement percentages by socio-economic groups and survey modality.

		Enumerator Administered	Mobile Phone Administered	Between modality comparison
Variable	Group	Mean Agreement (SD)	Mean Agreement (SD)	Statistical difference in agreement (P-Value)
Gender	Female	95.7 (4.26)	91.05 (7.99)	0.00^**^
	Male	95.1 (4.31)	91.23 (7.42)	0.01^*^
	*p-value*	0.34	0.16	
Age	18-24	96.1 (2.94)	93.26 (8.34)	0.59
	25-34	94.4 (4.69)	90.10 (8.65)	0.14
	35-44	96.6 (4.03)	91.60 (7.11)	0.00^**^
	Over 44	95.5 (4.13)	90.17 (5.84)	0.03^*^
	*p-value*	0.06	0.16	
Education	No school	96.8 (2.85)	93.1 (5.97)	1.00
	Primary school	95.8 (4.40)	90.6 (7.31)	0.00^***^
	Secondary school	93.8 (4.36)	90.6 (9.04)	1.00
	Post secondary	96.6 (3.45)	100 (-)	1.00
	Adult education	96.6 (-)	98.3 (2.44)	1.00
	No response	96.6 (3.45)	96.6 (-)	1.00
	*p-value*	0.08	0.21	
Ubudehe	Category 1	96.6 (4.08)	90.06 (7.60)	0.07
	Category 2	95.5 (4.01)	90.68 (7.54)	0.00^***^
	Category 3	95.0 (4.75)	91.81 (8.16)	1.00
	Category 4	–	–	–
	Category Don’t know	–	94.82 (7.31)	–
	*p-value*	0.54	0.88	

Note: P-values within modalities compare groups within each modality. P-values between modalities compare the same group across different modalities. ^*^p < 0.05, ^**^p < 0.01, ^***^p < 0.001

We found no significant differences within modalities based upon on when the DQQ was administered (e.g., morning, afternoon, evening) for either enumerator or mobile phone modality (S1 Table and S1 Fig) suggesting time of day has minimal impact on response variance within either modality.

### Diet quality indicators

In Table 4, we present the diet quality indicators generated from the observed and reported responses from the two modality groups. In only one case were reported data significantly divergent from the observed for enumerated administered respondents, with 18% of respondents reporting zero vegetable or fruit consumption compared to 8% observed (*p = 0.002*). Mobile-phone administered responses were significantly different from observed responses for two indicators: zero vegetable or fruit consumption (17% vs. 8%, *p = 0.003)* and NCD-Risk score (0.27 and 0.13, *p = 0.01*).

**Table 4:** Comparison of observed and reported diet quality indicators across reporting modality (enumerator: n = 150; and mobile-phone: n = 127).

			Enumerator Administered		Mobile- Phone Administered	
Diet Quality Indicator	Indicator Type	Range	Observed (SD)	Reported (SD)	Observed (SD)	Reported (SD)
MDD-W	Prevalence (%)	0-100	46.3 (50.0)	46.3 (50.0)	57.0 (49.8)	46.8 (50.0)
DDS	Score (Mean)	0-10	4.51 (1.43)	4.35 (1.61)	4.53 (1.41)	4.27 (1.83)
All-5	Prevalence (%)	0-100	8.67 (28.2)	11.3 (31.8)	4.72 (21.3)	11.0 (31.4)
NCD-Protect	Score (Mean)	0-9	3.49 (1.31)	3.33 (1.44)	3.55 (1.33)	3.39 (1.65)
NCD-Risk	Score (Mean)	0-9	0.12 (0.35)	0.08 (0.32)	0.13 (0.33)	0.27^*^ (0.66)
Zero Vegetable or Fruit	Prevalence (%)	0-100	8.00 (27.2)	18.0^**^ (38.5)	7.87 (27.0)	17.3^**^ (37.9)
Soft Drink	Prevalence (%)	0-100	2.0 (14.1)	3.3 (18.1)	3.15 (17.5)	5.5 (22.9)

* *p* < 0.05, ^**^p < 0.01, ^***^p < 0.001

Values in parenthesis are standard deviations. DDS: Dietary Diversity Score; DQQ: Diet Quality Questionnaire; MDD-W: Minimum Dietary Diversity for Women; NCD: non-communicable diseases; WFR: weighed food record.

### Costs

Costs per DQQ response are presented in Table 5. For the enumerator administered, the cost for enumerators, their accommodation, and other fixed costs were calculated across two data collection scenarios: i) where the DQQ is collected as a bespoke (standalone) survey, assuming 4 interviews per day; ii) where the DQQ is included as a module of an existing larger survey.

**Table 5:** Cost of administering the Diet Quality Questionnaire by enumerators and mobile phone. For enumerators, two scenarios of collection tested: where questionnaire is implemented as a standalone survey; where questionnaire is administered as a module within an existing larger survey.

Modality	Cost Breakdown	Cost ($USD)
Enumerator		
	Enumerator wage/day	25
	Enumerator accommodation/day	25
	Enumerator transport/day	10
	Enumerator phone communication budget/day	3
	Total	63
Enumerator Scenario		
	Standalone survey: Cost per DQQ (4 per day)	15.75
	Module within an existing survey: Cost per DQQ (6 minutes)	0.79
Mobile Phone		
	USSD system deployment	0.20
	USSD system administration	0.50
	Cost per DQQ response	0.70

DQQ: Diet Quality Questionnaire; USD: 2023 United States Dollar

The implementation of the DQQ as a bespoke standalone survey would cost $15.75 per response, whereas modular deployment, i.e., implementing the DQQ within an existing multi-topic survey, would cost $0.79, assuming completion within 6 minutes and that enumerators work for 8 hours. The cost for the mobile-phone administered DQQ was calculated at $0.70, or roughly 4% the cost of the standalone survey. The breakdown of these costs includes system (e.g., USSD session, SMS message to respondents) and administration (e.g., system maintenance). The system costs are per respondent, whereas the administration cost is fixed and would be the same for 10 respondents as 100,000, presenting the economy of scale benefits of mobile-phone deployment. The cost of data analysis is marginal, as standard code for data processing and quality control [40] and indicator calculation from DQQ data [37] can be run in less than 10 minutes for all responses.

## Discussion

In this study, we aimed to understand how, if at all, the method of data collection affects responses to the Diet Quality Questionnaire (DQQ). The DQQ administered by mobile phone produces comparable results to the DQQ administered by an enumerator, compared to an observed benchmark. Both methods were found to have high agreement with observed results, with enumerated responses having significantly higher agreement across the questions of the DQQ. A data cleaning protocol to identify (and remove) low quality responses improved agreement for both data collection modalities, but especially for mobile phone based collection. After removal, the two data collection modalities produced results with comparable accuracy, with the enumerated approach generating higher agreement levels (91% agreement vs 95% agreement, p = 0.05). These findings suggest that mobile-phone administration offers an accurate alternative to enumerator administered data collection.

In previous studies, demographic and economic factors have significantly influenced the accuracy of dietary recall methods and digital data collection systems [41,42,43]. Mobile-phone based systems are particularly sensitive to these factors, strongly associated with digital literacy of respondents [44,45]. Cross modality comparisons suggested significant differences in reporting, for age group, education level, and income category, aligning with previous studies [43, 46–50]. Modality accuracy was similar for both male and female respondents, within each modality, but significantly different across modalities with male and female respondents’ accuracy being significantly higher in enumerator-administered responses. Older (>35 years) mobile-phone respondents reported significantly less accurate responses, aligning with Grech et al. [43]. As with Couper and Kreuter [51], we observed that education level affected responses, with respondents educated to primary school level showing significantly lower agreement via mobile-phone. Only middle-income respondents (ubudehe category 2) provided significantly less accurate responses by mobile phone. We did not however find any significant differences within the same data collection modality (e.g., female-male reporting via mobile phones). Despite these variations, response accuracy did not drop below 90% in any socio-economic variable group, in either modality.

Our findings suggest the impact of digital literacy on response accuracy, with the least accurate responses tending to be generated by digitally marginalised groups. Identifying mechanisms to reduce digital barriers and improve response accuracy of marginalised respondents should be encouraged to bridge the accuracy gap and strengthen the viability of mobile-phone based data collection. In a recent usability study of the mobile-phone based system, Müller et al. [19] identified a number of modifications, derived from user design principles, to improve the user-experience of the system. Deployment of these modifications improved response quality from marginalised respondents [19]. Although reducing digital literacy marginalisation should be a priority, our findings suggest that complementary and tailored deployment of either modality could generate highly accurate repsonses.

Reviewing dietary indicators, we found that both mobile phone and enumerated respondents underreported consumption of fruits and vegetables, resulting in significantly higher reported ‘zero vegetable or fruit consumption’ prevalence in both groups (enumerated: *p* = 0.002; mobile-phone; *p* = 0.003). Reporting of consumption of ‘other vegetables’ was the least accurate of the DQQ questions, with high levels of false negative responses (underreporting). Reviewing the observed data, we found a considerable number of respondents (29%) consumed chayote (a member of the gourd family). This regionally prevalent food did not appear in the DQQ question relating to other vegetables, accounting for false negative responses and the zero vegetable or fruit consumption. Mobile-phone respondents were also found to significantly overreport NCD-Risk (*p* = 0.01). Further, the overreporting of NCD-Risk, in a country of rapid development and dietary transitions [52,53] could hint at indiscriminate aspirational responses, potentially encouraged by perceived anonymity of mobile-phone based data collection. These results suggest the limited impacts of individual response inaccuracy on population level dietary indicators and the differences between observed and reported outcomes.

Notwithstanding the omission of one commonly consumed vegetable item, it is notable that all other questions performed well even in a small subnational area, showing that in this context subnational adaptation needs were minimal. This study further reinforces the validity of the DQQ [30] and demonstrates its capacity for deployment and generation of accurate data across modalities, allowing for collection across spatial and temporal scales.

Cost comparisons reveal that when the DQQ is used as an additional module in an existing multi-topic survey, mobile-phone and enumerator administered data collection have similar costs ($0.70–0.79/respondent). However, for bespoke standalone collection of the DQQ, prices are much higher for enumerator ($16/respondent), but would still cost $0.7/respondent for mobile phone administered. There is much greater flexibility in using the mobile-phone modality, permitting rapid deployment without waiting for the timing of a multi-topic survey. Acceptance of the benefits of mobile phone use in data collection does come with a trade-off, reductions in response accuracy, which still remain high (>90%). However, in practical situations where data is outdated or budgets constrained, mobile phones could enable high-frequency and rapid data collection to understand seasonal changes, responses to shocks, and monitor the dynamism of dietary patterns.

### Limitations

Although this study has evidenced the potential of multi-modality collection of the DQQ and the opportunity for mobile-phone data collection to complement enumerators, a number of limitations should caveat the findings. Firstly, our response groups were not a representative sample of Musanze, nor of Rwanda. In particular, our study underrepresented the poorest and wealthiest income groups, yet we found that income group may have had a non-linear correlation on agreement between observed and reported data across modalities, which could be further tested in future studies. Secondly, respondents may have been more conscious of their consumption due to the weighed food record and the presence of enumerators, which may have positively influenced their recall in the DQQ. Finally, development and deployment of mobile-phone based technologies should be performed with concomitant frameworks for generating and collecting representative samples that also considered digital inclusion. Although we used a highly ubiquitous technology in Rwanda, we accept there are still considerable issues of digital inclusion, literacy, and equity that should be addressed in LMICs (e.g., [54]) and should be considered within the deployment of any self-administered data collection tool.

### Further improvement and next steps

Further improvement in data quality measures could generate a robust understanding of respondent quality and accuracy in situations where cross-validation (observation) is impossible. ‘In-situ’ metrics that monitor carelessness and (in)attentiveness to questions could provide an immediate filter for responses [18]. Respondents who answer affirmatively to a bogus question (e.g., “Did you eat penguin or kangaroo yesterday?”) or directed questions (“To show you are reading this question, please select ‘skip’.”) could be flagged. Addition of these embedded features would complement the post-hoc metrics deployed in this study and generate more robust measures of data quality. However, outlying ‘low quality’ responses will always exist, despite the modality and are an artefact of recall-based methods and mass data collection. We recommend testing of these data quality metrics for wider application to improve the robustness of mobile-phone data collection (e.g., [55]). Embedding of the data quality features and the findings of Müller et al. [19] have been made in an updated version of the self-administered data collection system which can now be deployed using USSD, WhatsApp, IVR or telegram, and currently deployed in Rwanda, Kenya, and Guatemala. 

## Conclusions

In this study we investigated how the modality of data collection affects responses to the Diet Quality Questionnaire (DQQ). Both mobile-phone and enumerator-administered modalities generated comparably high agreement rates between observed and reported data. However, agreement from mobile-phone respondents was lower by about 4 percentage points. Our findings partially reinforce the literature on digital literacy, with older respondents and those with primary education providing significantly less accurate responses using mobile-phones. Generated diet quality indicators from observed and reported data were comparable, suggesting that agreement differences had minimal impacts on population scale indicators. We found that mobile-phone and enumerated collection of the DQQ can, in certain circumstances, have similar costs of around $0.7/respondent, with mobile-phone collected data cheaper in all circumstances. Our findings underscore that, despite reductions in data accuracy, self-administered via mobile-phones is a viable complement to enumerator-administered data collection. Complementarity could be directed by target group characteristics, with data collection via mobile-phones deployed in regions or target populations with high digital literacy. Finally, widespread deployment of self-administered collection of dietary and food system data is already underway, greater adoption and intensity of collection seems inevitable. Ensuring mobile-phone based systems generate high-quality and accurate data is imperative.

## Supporting information

S1 Fig Agreement in responses to the Diet Quality Questionnaire for both modalities by socio-economic group. Responses by socio-economic groups within each modality group (enumerator: n = 150 and mobile-phone: n = 127). analysed as a function of agreement level between observed and reported responses. Boxes represent 25–75 percentiles, with median values displayed central lines, whiskers represent 5–95 percentile.
(DOCX)

S2 Fig Response agreement across data collection periods for both modalities. (enumerator: n = 150 and mobile-phone: n = 127) Boxes represent 25–75 percentiles, with median values displayed central lines, whiskers represent 5–95 percentile.
(DOCX)

S1 Table Relationship of observed-reported agreement rate with time of DQQ completion. (DOCX)

S1 Data Dataset from study (weighed food record and DQQ data). (XLSX)
