# Supplementary material for: Validating self-administration as an agile modality for high-frequency diet quality data collection
Source: PLoS One. 2025 Jun 25;20(6):e0317611. doi: 10.1371/journal.pone.0317611 (PMC12193772; doi:10.1371/journal.pone.0317611)
Supplement: S1 Fig — Responses by socio-economic groups within each modality group (enumerator: n = 150 and mobile-phone: n = 127). analysed as a function of agreement level between observed and reported responses. Boxes represent 25–75 percentiles, with median values displayed central lines, whiskers represent 5–95 percentile. (DOCX) [file pone.0317611.s001.docx]

**Supplementary Information**

**Fig S1.** Agreement in responses to the Diet Quality Questionnaire for both modalities of reporting (enumerator: n=150 and mobile-phone: n=127). Responses by socio-economic groups within each modality group analysed as a function of agreement level between observed and reported responses. Boxes represent 25-75 percentiles, with median values displayed central lines, whiskers represent 5-95 percentile.
