# Supplementary material for: Validating self-administration as an agile modality for high-frequency diet quality data collection
Source: PLoS One. 2025 Jun 25;20(6):e0317611. doi: 10.1371/journal.pone.0317611 (PMC12193772; doi:10.1371/journal.pone.0317611)
Supplement: S2 Fig — (enumerator: n = 150 and mobile-phone: n = 127) Boxes represent 25–75 percentiles, with median values displayed central lines, whiskers represent 5–95 percentile. (DOCX) [file pone.0317611.s002.docx]

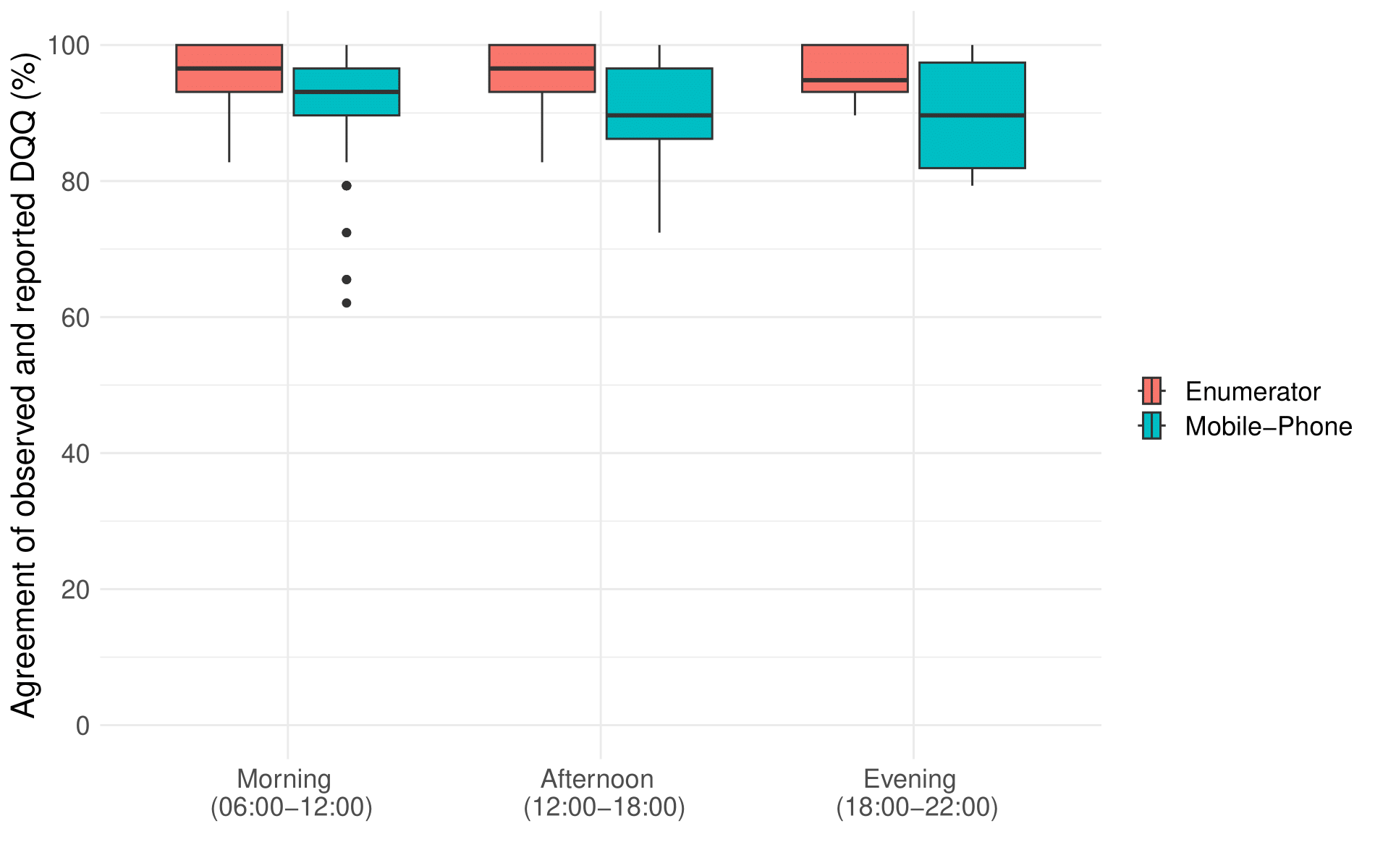


**Fig S2.** Response agreement across data collection periods for both modalities of reporting (enumerator: n=150 and mobile-phone: n=127) Boxes represent 25-75 percentiles, with median values displayed central lines, whiskers represent 5-95 percentile.
