# Supplementary material for: Validating self-administration as an agile modality for high-frequency diet quality data collection
Source: PLoS One. 2025 Jun 25;20(6):e0317611. doi: 10.1371/journal.pone.0317611 (PMC12193772; doi:10.1371/journal.pone.0317611)
Supplement: S1 Table — (DOCX) [file pone.0317611.s003.docx]

**Table S1.** Relationship of observed-reported agreement rate with time of DQQ completion.

|  |  | Enumerator Administered | Mobile Phone Administered | Between modality comparison |
| --- | --- | --- | --- | --- |
| Variable | Group | Mean Agreement (SD) | Mean Agreement (SD) | Statistical difference in agreement  (P-Value) |
| Sex | Morning | 95.7  (4.14) | 91.3  (7.99) | 0.00** |
|  | Afternoon | 95.1  (4.44) | 90.7  (6.58) | 0.01* |
|  | Evening | 95.5  (4.32) | 89.7  (6.58) | 0.26 |
|  | *p-value* | 0.69 | 0.60 |  |
